# All‐Inkjet‐Printed 3D Alveolar Barrier Model with Physiologically Relevant Microarchitecture

**DOI:** 10.1002/advs.202004990

**Published:** 2021-03-08

**Authors:** Dayoon Kang, Ju An Park, Woojo Kim, Seongju Kim, Hwa‐Rim Lee, Woo‐Jong Kim, Joo‐Yeon Yoo, Sungjune Jung

**Affiliations:** ^1^ School of Interdisciplinary Bioscience and Bioengineering Pohang University of Science and Technology (POSTECH) 77 Cheongam‐Ro, Nam‐Gu Pohang 37673 Korea; ^2^ Department of Convergence IT Engineering Pohang University of Science and Technology (POSTECH) 77 Cheongam‐Ro, Nam‐Gu Pohang 37673 Korea; ^3^ Department of Mechanical Engineering Pohang University of Science and Technology (POSTECH) 77 Cheongam‐Ro, Nam‐Gu Pohang 37673 Korea; ^4^ Department of Life Sciences Pohang University of Science and Technology (POSTECH) 77 Cheongam‐Ro, Nam‐Gu Pohang 37673 Korea; ^5^ Department of Materials Science and Engineering Pohang University of Science and Technology (POSTECH) 77 Cheongam‐Ro, Nam‐Gu Pohang 37673 Korea

**Keywords:** 3D cultures, air‐blood barrier, bioprinting, influenza A virus, lungs

## Abstract

With the outbreak of new respiratory viruses and high mortality rates of pulmonary diseases, physiologically relevant models of human respiratory system are urgently needed to study disease pathogenesis, drug efficacy, and pharmaceutics. In this paper, a 3D alveolar barrier model fabricated by printing four human alveolar cell lines, namely, type I and II alveolar cells (NCI‐H1703 and NCI‐H441), lung fibroblasts (MRC5), and lung microvascular endothelial cells (HULEC‐5a) is presented. Automated high‐resolution deposition of alveolar cells by drop‐on‐demand inkjet printing enables to fabricate a three‐layered alveolar barrier model with an unprecedented thickness of ≈10 µm. The results show that the 3D structured model better recapitulate the structure, morphologies, and functions of the lung tissue, compared not only to a conventional 2D cell culture model, as expected, but also a 3D non‐structured model of a homogeneous mixture of the alveolar cells and collagen. Finally, it is demonstrated that this thin multilayered model reproduce practical tissue‐level responses to influenza infection. Drop‐on‐demand inkjet‐printing is an enabling technology for customization, scalable manufacturing, and standardization of their size and growth, and it is believed that this 3D alveolar barrier model can be used as an alternative to traditional test models for pathological and pharmaceutical applications.

## Graphical abstract

The morphological, functional, and microenvironmental features of human alveolar barrier tissue are recapitulated by providing uniform and continuous cell–cell contact sites and cell–matrix interaction in a carefully controlled 3D microarchitecture. To this end, high‐resolution inkjet bioprinting was used to reconstitute four essential alveolar cell types in 3D multilayered architecture. This model reproduced practical tissue‐level responses to influenza infection.

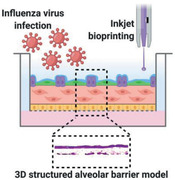

## 1 Introduction

The lungs are the major organs of respiration that is most vulnerable to infection and injury due to its continual exposure to fine particles, harmful chemicals, and infectious organisms from the external environment. The pulmonary alveolar of the human lung have a three‐layered structure with epithelium and endothelium located on either side of a basement membrane. The vital air–blood interface with a thickness of only a few micrometers occupies a large area of ≈100–140 m^2^.^[^ ^1^, ^2^ ^]^ Lung epithelium is composed of two main cell types: type I and type II cells. Squamous type I alveolar cells (AT I) are involved in gas exchange process between the alveoli and blood. Cuboidal type II alveolar cells (AT II), one of the primary targets for influenza A pneumonia, secrete pulmonary surfactant and participate in innate immune response of the lung.^[^ ^3^ ^]^ Although the population of AT I cells is only half compared to AT II cells, yet the flattened cells constitute more than 90% of the alveolar surface area.^[^ ^4^, ^5^ ^]^ An alveolus also includes fibroblasts and extracellular matrix (ECM), a component of the basement membrane, and microvascular endothelial cells that constitute capillaries.^[^ ^6^ ^]^

There is an urgent need for morphologically and functionally relevant, yet reliably manufacturable and customizable models of human lung tissue to study disease pathogenesis, drug efficacy, and toxicology.^[^ ^7^ ^]^ Various methods have been used to fabricate biomimetic *in vitro* 3D alveolar barrier tissue models. The *in vitro* air–blood barrier model produced by Bengalli et al. was fabricated on a porous membrane using a conventional pipetting method.^[^ ^8^ ^]^ Their *in vitro* model contributed to investigation of the effects and the toxicity of inhaled nanoparticles. With the development of microfluidic technology, research on organ‐on‐a‐chip has also been conducted.^[^ ^9^ ^]^ A representative lung‐on‐a‐chip by Huh and colleagues successfully recreated the mechanical properties as well as structural aspects of the alveolar barrier.^[^ ^10^, ^11^ ^]^ To develop a micro‐engineered lung model, they used soft lithography‐based microfabrication techniques to build a microchannel system with a microporous polymer membrane. The formation of an alveolar–capillary interface was achieved by seeding epithelial and endothelial cells through tubing. More recently, 3D bioprinting was applied to mimic alveolar barrier structures.^[^ ^12^ ^]^ Horváth et al. used valve‐based bioprinting to fabricate an *in vitro* model consisting of lung epithelial and endothelial cell lines and compared it with a model produced using manual pipetting. However, the difficulty of *in vitro* lung tissue modeling still lies in the challenge to recapitulate the extremely thin and layered architecture and to control cell–cell and cell–extracellular matrix communication interactions by spatial arrangements of multiple cell types composing the tissue within the unique structure.

Meanwhile, bioprinting technology is one of the emerging technologies that can be used to fabricate a 3D tissue model of a complex structure. Bioprinting enables an automated deposition of cells and biomaterials in 3D manner for highly controlled and customized production of tissue models.^[^ ^13^, ^14^ ^]^ To date, bioprinting techniques have been applied to various tissues including skin, cornea, retina, liver, blood vessels, airways, bone cartilage, heart, and bladder.^[^ ^15^, ^16^, ^17^, ^18^, ^19^, ^20^ ^]^ Tissue engineering with bioprinting can open the way for more accurate models for *in vitro* drug screening and toxicity research. Among bioprinting technologies, piezo‐electric inkjet bioprinting is most suitable to reconstitute the characteristics of thin and spatially complex soft tissues. It is because the drop‐on‐demand printing method has advantages over other bioprinting techniques such as high resolution, high printing speed, high cell viability, and low material waste.^[^ ^21^, ^22^, ^23^ ^]^ The inkjet‐based bioprinter includes a piezoelectric actuator in its nozzle, which generates acoustic waves inside an ink chamber upon an electric pulse to eject very small droplets with a typical volume of 1–100 pL (10^−12^ L). This capability of inkjet printing has been proved to enable one to micropattern the living mammalian cells in 2D and 3D environments with high accuracy and speed.^[^ ^24^, ^25^, ^26^, ^27^ ^]^

Here, we report an all‐inkjet‐printed alveolar barrier model with the functional layers of epithelium and endothelium with a collagen basement membrane in‐between. High‐resolution patterning of four types of human alveolar cells, namely, NCI‐H1703 (alveolar epithelial type I‐like cells), NCI‐H441 (alveolar epithelial type II cells), HULEC‐5a (lung microvascular endothelial cells), and MRC5 (lung fibroblasts) along with type I collagen, allowed us to recapitulate the structural and functional complexity of alveolar tissue with a thickness of the order of 10 µm. Measurements of transepithelial/endothelial electrical resistance (TEER), permeability tests, histological and immunohistochemical analysis, and quantitative real‐time polymerase chain reaction (qPCR) were performed to evaluate the structural and functional characteristics of the all‐printed 3D model. Finally, the model was infected with influenza virus and its potential as a viral infection model was demonstrated.

## 2 Results

### 2.1 Inkjet Bioprinting of Alveolar Barrier Models

**Figure 1:**
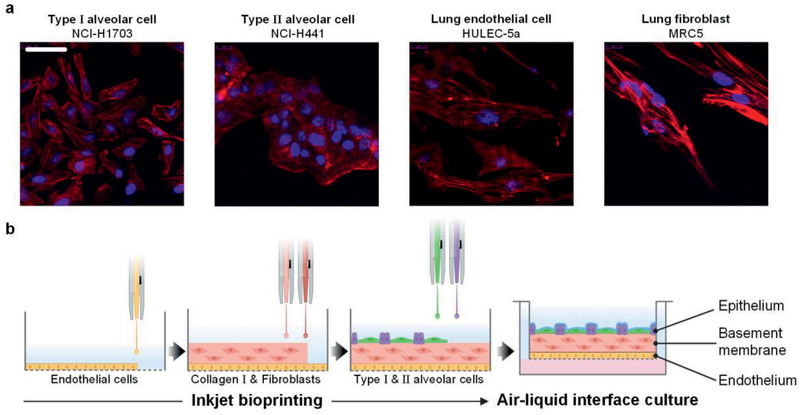
Fabrication of alveolar barrier model a) Confocal fluorescence microscopy images of human alveolar cell lines stained with phalloidin. F‐actin was stained red and nuclei was stained blue. Scale bar: 50 µm. b) Schematic diagram of inkjet‐printing process for fabrication of a 3D alveolar barrier model. All cells were inkjet‐printed in a layer‐by‐layer manner. This image was created using BioRender (https://biorender.com/).

**Figure** **1** shows the types of alveolar cell lines and the inkjet printing process for the fabrication of an ultrathin, three‐layered alveolar barrier model. As model cells, we used the human lung cell line NCI‐H1703 as flat squamous AT I, NCI‐H441 as cuboidal AT II, endothelial HULEC‐5a, and MRC5 as fibroblasts. The confocal fluorescence microscopy images in Figure 1a show the morphologies of each cell type. The fabrication of a thin, three‐layered alveolar barrier model was accomplished via the precise inkjet printing of these cells and collagen (Figure 1b). In a Transwell permeable support, endothelial cell ink, collagen ink, and fibroblast‐containing collagen ink were sequentially printed in a layer‐by‐layer manner. The endothelial cell ink was first inkjet‐printed onto the porous membrane of the Transwell insert. After incubation for 24 h, the collagen and the fibroblast‐laden collagen inks were printed on top of the endothelial layer. After the collagen had been fully crosslinked for 24 h in an incubator, the AT I and AT II epithelial cells with the ratio of 1:2 in number were uniformly deposited on the underlying layer. All the printing process was performed at room temperature, except the collagen‐based inks which was printed at 4 °C to prevent them from crosslinking during jetting. The all‐inkjet‐printed alveolar model was cultured for 14 days with the epithelium exposed to air, and then its structure and physiological functions were further evaluated and analyzed.

### 2.2 Optimization of Inkjet Cell‐Printing Process

**Figure 2:**
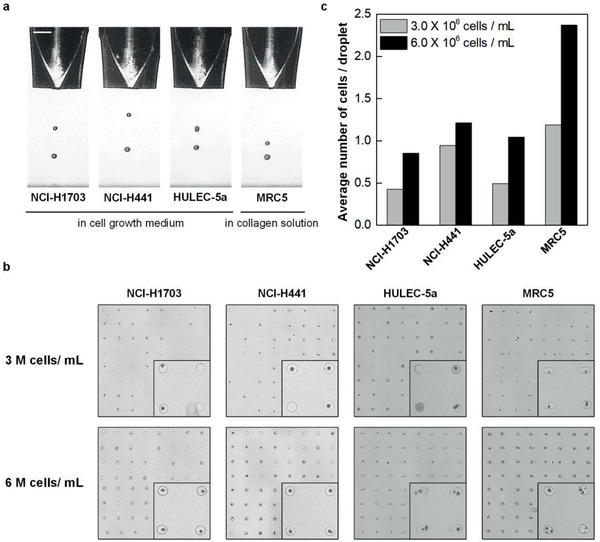
Optimization of inkjet‐bioprinting parameters. a) Single‐flash images of inkjet‐printing of human alveolar cells with a piezoelectric nozzle with a diameter of 80 µm. Scale bar: 100 µm. b) Representative images of 8 × 8 arrays of cell‐laden droplets printed on a glass substrate with a 500‐µm spacing. Inset: magnified 2 × 2 arrays. c) The average number of cells per a droplet in 32 × 32 arrays.

First, we developed a reliable inkjet process for each cell type. The bio‐inks for NCI‐H1703, NCI‐H441, and HULEC‐5a cells were prepared in cell growth medium and MRC5 cells were suspended in a diluted collagen ink. **Figure** **2**a shows single‐flash jetting images of the bio‐inks. All the bio‐inks were ejected with an 80‐µm‐sized piezoelectric nozzle at a jet speed of ≈3 m s^−1^. A precisely adjusted bipolar actuation waveforms and pneumatic pressures enabled the patterning of cell‐laden drops with high resolution and stability. Its rise, fall, and dwell times were set to around 10 µs and the peak drive voltage was ±80 V in all of the experiments.

Next, we examined the number of printed cells in a single pL‐level droplet to manipulate the accurate number of cells deposited on each layer of alveolar tissue. To this end, we printed an array of 32 × 32 droplets on a glass substrate for each cell type in concentrations of 3 × 10^6^ and 6 × 10^6^ cells mL^−1^. Figure 2b shows the representative 8 × 8 images of the printed cells. The average number of cells ejected with a drop is presented for each cell type in Figure 2c. In the magnified 2 × 2 array images, cell growth medium ink had fewer cells in the droplets than collagen ink. Upon counting the number of cells carried on average on each droplet (Figure 2c), we found that approximately a single cell was printed in a droplet when the concentration was 6 × 10^6^ cells mL^−1^ in cell growth medium ink. For the diluted collagen ink, a single cell was printed at the concentration of 3 × 10^6^ cells mL^−1^. We determined the concentration of each cell ink based on these conditions to fabricate 3D alveolar models with high resolution and accuracy.

### 2.3 Viability Evaluation of Inkjet‐Printed Cells

**Figure 3:**
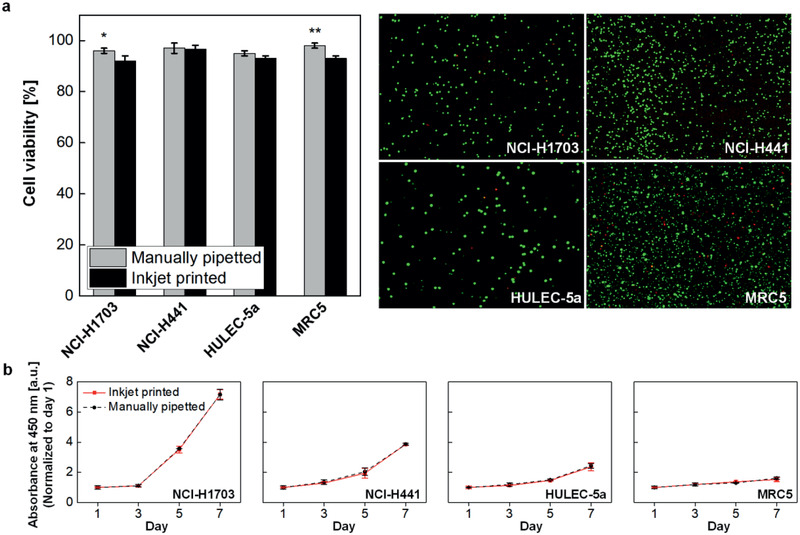
Post‐printing cell viability test. a) The LIVE/DEAD assay was performed to determine viability of cells immediately after printing, (*n* = 3). b) The CCK‐8 assay was performed to evaluate cell proliferation for 7 days after printing, (*n* = 3).

To investigate any potential influence of jetting through a micron‐sized nozzle on cell viability and proliferation, we performed both LIVE/DEAD assay immediately after printing and CCK‐8 assay for 7 days. **Figure** **3**a shows cell viability obtained by analyzing cell populations stained with calcein AM (green, live cells) and ethidium homodimer‐1 (red, dead cells). The results show high cell viability of >90% in the printed groups, which were close to the values of manually pipetted control groups. This high survival rate was consistently found for all the human cell types used in this work. Figure 3b presents the proliferation rates of the printed and the control groups for each cell line over a week. During the experiments, any significant cell death was not observed with the printed cell groups well following the growth trend of the pipetted control group, demonstrating that inkjet bioprinting did not adversely affect the alveolar cells in use.

### 2.4 Structural Characteristics and Barrier Properties of Printed Alveolar Models

**Figure 4:**
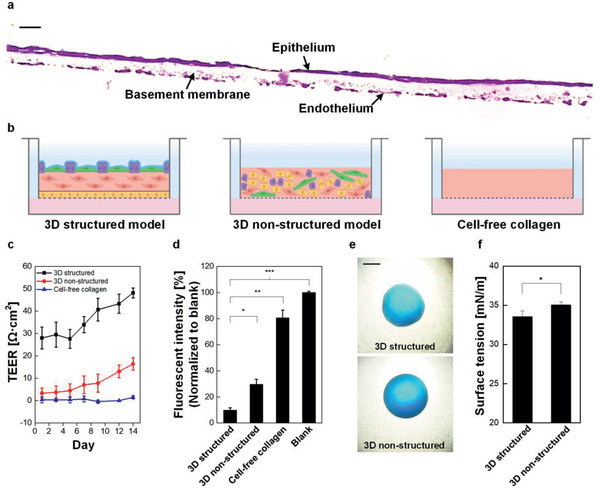
Evaluation of structure and barrier properties of alveolar barrier models. a) A cross‐sectional image of alveolar barrier model was stained with hematoxylin & eosin. Scale bar: 20 µm. b) Schematic of three model groups. The number of cells and the volume of collagen were identical across the groups. This image was created using BioRender (https://biorender.com/). c) Comparison of TEER and d) permeability between models (*n* = 3). fluorescence intensity was normalized to blank as Transwell insert without cells. e) Microscopic images of droplets of a mixture of dimethylphthalate and 1‐octanol on apical surfaces of a 3D structured and a 3D non‐structured model. Scale bar: 1000 µm. f) Comparison of surface tension between 3D structure and 3D non‐structured models, (*n* = 3).

We fabricated very thin three‐layered alveolar models, using the inkjet printing process developed for the four alveolar cell types. The histological image in **Figure** **4**a clearly shows its three‐layered structure with homogeneous distributions of epithelial and endothelial cells on both sides of a collagen basement membrane. Some minor damage to the thin endothelial layer was likely to occur during frozen section procedure. The unprecented thickness of the inkjet‐printed alveolar model was measured to be 12 ± 1.1 µm by using a high‐resolution stylus profiler before tissue fixation and then freezed to avoid morphological distortion during the cryosection (Figure S1, Supporting Information). Sub‐10‐µm thick models could be achieved by controlling the volume of printed drops, but we did not manage to obtain cryosection images for them due to tissue brittleness.

To determine the alveolar barrier properties, we conducted TEER and permeability measurements for the 3D structured model. For comparison, we evaluated together with a 3D non‐structured collagen model containing a heterogeneous mixture of all four cell types and a cell‐free collagen model without cells as control groups (Figure 4b). The physical dimensions, the number of cells, and the fabrication conditions for the 3D structured model remained unchanged to fabricate the controls. The results showed a distinct difference between our model and the control groups, demonstrating our structured tissue formed tighter junction than the other models. As can be seen in Figure 4c, the TEER value of the 3D structured model had higher value from the day 1 and increased steadily for the following 14 days, whereas the changes in the 3D non‐structured model remained smaller for the same period. The cell‐free collagen model showed no barrier formation with resistance values of ≈0 Ω cm^2^ as expected. The relatively lower TEER values over previous studies using NCI‐H441 cells^[^ ^28^, ^29^ ^]^ may be attributed to the slight contraction of the collagen membrane of our model during maturation or the different culture conditions. Furthermore, we carried out permeability tests by measuring fluorescence of fluorescein isothiocyanate‐labeled dextran (FITC‐dextran) from a basolateral side (Figure 4d). The permeability of the 3D structured model was significantly lower than those of the control groups due to homogeneous distribution and 3D stacking of cells without voids during printing process. These results identify the formation of tight junctions in the printed three‐layered intercellular models.

Apical surface properties of the epithelial layer were further characterized by drop spreading technique for surface tension measurement according to the protocol in previous studies. ^[^ ^30^, ^31^ ^]^. The pulmonary surfactant plays an important role in maintaining the structural integrity of the respiratory epithelium by lowering surface tension. A droplet of a mixture of dimethyl phthalate and 1‐octanol with a diameter (*D*_o_) of 1.4 mm was placed on the surface of a 3D‐structured model and a 3D‐non‐structured model, and its spreading diameter (*D*) on the surface was measured under a microscope (Figure 4e). The relative drop diameter (*D*/*D*_o_) was then converted into values of surface tension which gives 33.6 mN m^–1^ for the 3D structured model and 35.1 mN m^–1^ for 3D non‐structured model (Figure 4f). We believe that the decrease in surface tension was attributable to effective secretion of surfactant proteins in the three‐layered structure.

### 2.5 Immunohistochemical Analysis

**Figure 5:**
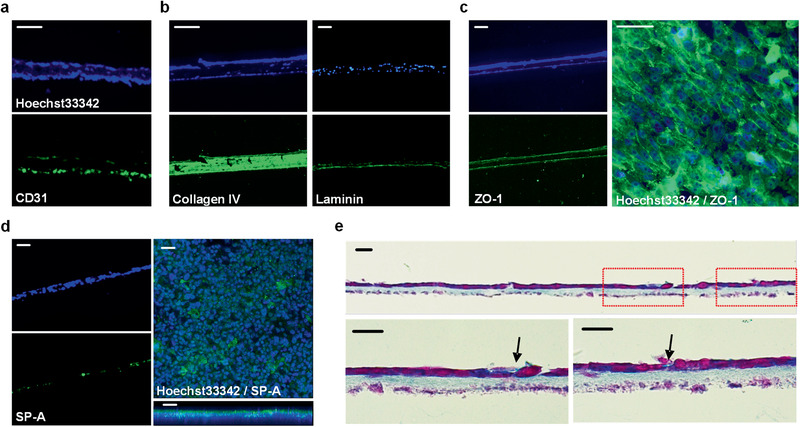
Structural and functional characterization of 3D inkjet‐printed alveolar barrier model. a–d) Immunofluorescence microscopic images using specific antibodies (green) and nuclei staining reagent Hoechst33342 (blue); the endothelial marker CD31 derived from HULEC‐5a cells (a), the basement membrane constituent collagen IV and laminin resulted from cell‐ECM interaction (b), the tight junction protein ZO‐1 which presents a solid intercellular connection (c), and hydrophilic surfactant SP‐A secreted by type II alveolar cells (d). Scale bars: 50 µm. e) Microscopic cross‐section image after Alcian blue and nuclear fast red staining. Nuclei in cells and the secreted surfactant are stained in purple and light blue, respectively. The arrows indicated newly produced surfactant in the epithelium layer. Scale bars: 20 µm.

Immunohistochemistry was performed to investigate the structural composition and the function of the alveolar barrier. With Hoechst 33342, location of the four‐type cells within the model was visualized. CD31 antibody was examined to verify the microvascular density of the alveolar barrier models, and the endothelial adhesion marker was highly expressed in the endothelium layer (**Figure** **5**a). Collagen IV and laminin antibody were also tested to demonstrate the distribution of components in the basement membrane (Figure 5b). We observed that type IV collagen was synthesized at a high level inside the basement membrane, while laminins were expressed along the boundary of the basement membrane in contact with the endothelium and epithelium. The proper deposition of laminins indicated that the endothelial and epithelial cells were aligned at the basement membrane with a polarity.

Furthermore, to determine the tight junction formation of the alveolar barrier, immunostaining of tight junction protein ZO‐1 was performed (Figure 5c). It was distinctly expressed on both sides of endothelium and epithelium. The abundant tight junction in epithelium of the alveoli played significant roles by separating the air‐exposed compartment of the respiratory system from the aqueous interstitial compartment efficiently. Top‐view image of the epithelium surface also confirmed that the tight junction was highly expressed over the entire area. Finally, we investigated the surfactant secretion function of AT II cells in the epithelium by immunohistochemical staining for surfactant protein A (SP‐A) and by Alcian blue staining (Figure 5d,e). The top‐view and side‐view images of the apical surface showed a thin film containing the pulmonary surfactant that lines the gas‐exchanging surface of the pulmonary epithelium (Figure 5d). Figure 5e presents the Alcian blue staining of the surfactant with light blue around the cubic cells of epithelium. These histological analysis verified that the printed alveolar construct effectively maintained its structure of three layers with their appropriate functions.

### 2.6 Expression of Functional Genes in Alveolar Barrier Model

Next, we performed qPCR tests to quantify the levels of representative genes and investigated cellular functions of our model. We chose alveolar‐specific genes, which express tight junction and adherence junction proteins (ZO‐1, Occludin, and E‐cadherin), ion channel proteins (*α*‐, *β*‐, *γ*‐ENaCs and ATP1A1), and surfactant proteins (SP‐A and SP‐B). 3D non‐structured models and 2D cell culture models, both with a mixture of four types of alveolar cells, were also tested to be compared with our 3D structured model.

**Figure 6:**
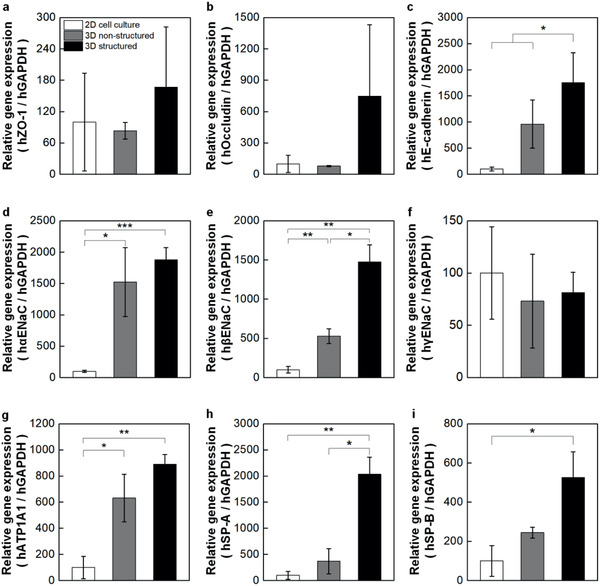
Comparison of representative alveolar gene expression profiles between conventional and 3D inkjet‐printed structured models. Relative mRNA expression was measured via qPCR for 2D cell culture, 3D non‐structured, and 3D structured models using specific markers involved in a–c) intercelluar junction formation, d–g) epithelial ion channels and ion transport, and h,i) pulmonary surfactant secretion. Each expression levels were normalized by the level of 2D cell culture models. (*n* = 12, 9, and 13 for 2D cell culture, 3D non‐structured, and 3D structured models, respectively. The expression level of each gene was normalized by the value at expression level of 2D cell culture group.)

For the tight junction proteins, mRNA levels of ZO‐1 and Occludin were higher in the 3D structured model than the other models (**Figure** **6**a,b). The more distinct difference among the models was observed for adherence junction protein with its level in the 3D structured model 18‐fold higher than that in the 2D cell culture model (Figure 6c). These results confirm tighter barrier characteristics of the 3D structured model over the other models, which agrees with the results from the TEER measurements (Figure 4c). The evaluation of epithelial sodium channels (*α*‐ and *β*‐ENaCs) and Na^+^/K^+^‐transporting ATPase subunit *α* 1 (ATP1A1) showed the same trend with the highest mRNA expressions in 3D structured model and the lowest in 2D cell culture model, whereas negligible difference was found in *γ*‐ENaC expressions (Figure 6d–g).

We further investigated the mRNA expression of major alveolar surfactant proteins. We chose hydrophilic SP‐A which has innate immune functions against respiratory pathogens such as influenza A virus and hydrophobic SP‐B essentially required for breathing.^[^ ^32^ ^]^ As seen in Figure 6h,i, marked differences were observed among the groups with the highest gene expressions in the 3D structured model. As expected, classical 2D‐grown monolayers of alveolar cells resulted in lower expression levels in most of the genes compared to in 3D microenvironments. It is noteworthy that the results demonstrate the dependency of genes that promoted growth in a 3D‐specific context on its microenvironment structure.

### 2.7 Influenza Virus Infection and Analysis of Antiviral Responses in 3D Structured Models

**Figure 7:**
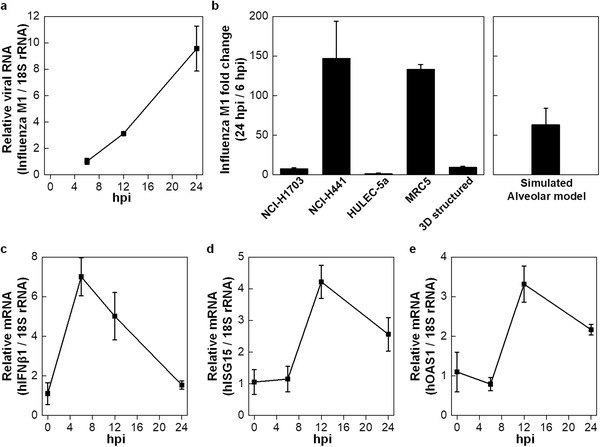
PR8 influenza virus infection and analysis of anti‐viral responses. a) 3D structured models were infected with influenza A virus (A/PR8/1934(H1N1), 10 TCID_50_mL^–1^) for the indicated times (6‐, 12‐, and 24‐hour post infection). The expression of Influenza M1 viral RNAs were analyzed via qPCR. b) Each cell type was infected with influenza A virus (A/PR8/1934(H1N1), 10 TCID_50_mL^–1^) for the 6 and 24 h. Left, proliferative kinetics (M1 at 24 hpi / 6 hpi) of the intracellular influenza viral genome (M1) were measured in 3D structured model (from (a)) and each cells by calculating the fold change of influenza M1 between intracellular viral RNA at 24 hpi versus that at 6 hpi. Right, artificial value of cell mixture calculated based on viral propagation fold of each cell types. c–e) The expression level of human IFNβ1 (c), human ISG15 (d), and human OAS1 (e) mRNAs at alveolar barrier model (from (a)) were analyzed via qPCR. (*n* = 3).

Both the upper and lower parts of the respiratory tract are common target sites for respiratory infectious diseases caused by respiratory viruses such as influenza A virus. Although the majority of influenza A virus infections occur in the upper respiratory tract, its infection in the lower respiratory tract often results in severe pneumonia accompanied by damage to the alveolar structure.^[^ ^33^ ^]^ We therefore assessed whether influenza A virus infection and the antiviral responses against infection could be properly observed in our alveolar barrier model. A total of 10 TCID_50_ mL^–1^ of seasonal H1N1 influenza A viruses (PR8 strain) were applied to the air side of the alveolar barrier models to mimic infection with influenza in the epithelial layers of the respiratory tract and the proliferative kinetics of the intracellular influenza viral genomes at 6‐, 12‐, and 24‐hour post‐infection (hpi) (**Figure** **7**a). The amount of total intracellular influenza viral genomes continuously increased, with maximum proliferation observed at 24 h after infection. Relative to 6 hpi, about 10‐fold higher levels of intracellular virus were detected at 24 hpi using the alveolar barrier models. To compare the susceptibility to virus infection of the alveolar barrier model, 10 TCID_50_ mL^–1^ of H1N1 influenza A virus infected the same number (1.0 × 10^6^) of NCI‐H1703, NCI‐H441, HULEC‐5a, and MRC5 cells after individually culturing in 2D environment. Their proliferative kinetics of the intracellular influenza viral genomes was then measured in each cell type. Both NCI‐H441 and MRC5 cells were extremely susceptible to influenza infection, with 147.2‐ and 133.5‐fold induction at 24 hpi relative to the levels at 6 hpi (Figure 7b). In contrast, NCI‐H1703 and HULEC‐5a cells were less susceptible to infection, with 7.7‐ and 1.5‐fold induction at 24 hpi relative to the level at 6 hpi.

Since different amounts of each cell type were used to generate the alveolar barrier models, the virus production rate of each cell type was numerically calculated and proportionally added (Figure 7b, right). In contrast to 9.5‐fold induction of intracellular virus observed in the real alveolar barrier models, more than 63.3‐fold induction obtained from simulated model. The antiviral responses of the infected alveolar barrier model were then examined by monitoring the production of type I interferon (IFN) and the expression of IFN‐stimulated gene 15 (ISG15) and the 2′–5′‐oligoadenylate synthetase 1 (OAS1) which are among the most important components of the immune system induced by IFN, at 6, 12, and 24 h after infection. Although active viral proliferation was observed at 24 h after infection, both type I IFN and ISGs expression levels had dramatically declined at this time point (Figure 7c–e). These results indicate that our alveolar barrier model is not just a mixture of diverse cells but is a structural unit that serves immune functions.

## 3 Discussion

In this study, we aimed to recapitulate the morphological, functional, and microenvironmental features of human alveolar barrier tissue by providing uniform and continuous cell–cell contact sites and cell–matrix interaction in a carefully controlled 3D microarchitecture. To this end, we used high‐resolution inkjet bioprinting to reconstitute four essential alveolar cell types in 3D multilayered architecture. One critical challenge in 3D tissue modeling technologies is their inability to pattern various cell types with micron accuracy, which limits its applications in mimicking complex multicellular microenvironment. Our automated drop‐on‐demand patterning process enabled the precise control of spatial arrangements and populations of multiple cell types in layer by layer, which allowed us to reproduce intricate microarchitecture and morphologies as well as its major functions such as barrier integrity and surfactant secretion. The measurements of the thickness through the stylus surface profiler and histological images confirmed that the model had a uniform thickness of ≈10 µm, which is the thinnest *in vitro* model to date, to the best of our knowledge. The histological and immunohistochemical analysis in Figures 4a and 5 showed that even in this thin structure, the epithelial and endothelial cells in our model were well arranged as designed and they were clearly separated by a fibroblast‐containing collagen basement membrane. This contrasts with the most of the previous *in vitro* lung models, which used relatively thick synthetic porous membranes.^[^ ^8^, ^10^, ^12^ ^]^

There is no doubt on the significance and urgency to develop structurally and physiologically more relevant 3D *in vitro* lung models for drug screening, disease modeling, and toxicity prediction. Cell‐based assays using 2D cellular monolayers do not fully mimic the *in vivo* situation where cells grow and interact in a 3D microenvironment.^[^ ^34^ ^]^ As expected, our data on protein expression in Figure 6 well agree with this, indicating significant differences between cells grown in 2D and 3D culture models. Currently, many of the 3D epithelium models mainly consist of entrapped cells, often a homogeneous mixture of multiple cell types, within a gel, being lack of control over the dynamic spatial cell microenvironment in the 3D construct. This also does not fully re‐establish physiological interactions of cell–cell and cell–matrix. For a tissue such as the alveolar barrier consisting of multiple layers, providing epithelium‐fibroblast and endothelium‐fibroblast interactions is key to the development of actual lung model since the maturation and function of epithelium and endothelium are affected by a fibroblast‐secreted mediators.^[^ ^35^, ^36^, ^37^ ^]^ Studies shows that their abnormal interactions contribute to the development and progress of pulmonary diseases.^[^ ^38^, ^39^, ^40^, ^41^ ^]^

Together with the comparison between 2D and 3D models, we investigate the difference between the 3D‐printed model with multilayer microarchitecture and the 3D non‐structured co‐culture model produced by homogeneous seeding alveolar cells in a collagen mass. Despite the identical components in these two models such as the number of cells and the volume of collagen matrix, the results in Figures 4c,d and 6a–c demonstrate that our 3D structured model shows better barrier integrity with the higher gene expression levels of intercellular junction proteins over the 3D non‐structured counterpart. The difference was found in surfactant secretion. We identified a surfactant protein of SP‐A through the immunohistological analysis of the 3D structured model (Figure 5d). Further investigation on gene expression profiles reveals the higher expression of pulmonary surfactant markers (SP‐A and SP‐B) for the 3D structured model, which well agrees with the results on the measurement of surface tension in Figure 4e,f. Furthermore, the gene expression of ion channels and transport proteins follows the same trend, showing distinctively upregulated mRNA expressions in the ion channels and transport proteins of the 3D structured model, compared to the conventional model.

Unlike the 3D non‐structured model where all the four types of cells spread homogeneously in collagen matrix and grow in a 3D environment, each type of cells interacts with their surroundings in a layered structure with the entire epithelial monolayer exposed to air throughout the culture process in the 3D structured model. Therefore, we believe that the reconstitution of the 3D spatial arrangements even within this extremely thin construct made a significant impact on their physiological functions.

The recapitulation of the physiological functions of barrier properties, surfactant secretion, and ion transport *in vitro* allowed us to apply our model to influenza A virus infection and initial immune responses which cause severe pulmonary diseases. It is the only influenza virus to cause flu pandemics and claims ≈250,000 to 500,000 lives annually worldwide. In particular, the emergence of pandemic influenza A virus strain H1N1 in 2009 resulted in ≈60.8 million cases and 12,469 deaths in the United States only for 1 year.^[^ ^42^ ^]^ In this work, the 3D model successfully demonstrates the continuous increase in viral RNA up to 24 h after virus infection and corresponding anti‐viral responses (Figure 7). These findings suggest that alveolar barrier fabrication may provide a reliable 3D‐model system to investigate the intercellular and intracellular kinetics of viral infection and the antiviral responses against it.

We demonstrate that our inkjet‐bioprinting fulfils the key requirements in generating an *in vitro* 3D alveolar barrier model. One limitation of the model is on its rigid flat shape, despite its 3D structure. A pulmonary alveolus has the form of a hollow cup‐shaped cavity, which is exposed to physiologically 5% of elongation with inhaled air, which passes through an alveolar duct connected to alveolar sacs.^[^ ^43^ ^]^ If this structure and function is overcome in future work, the inkjet‐printed *in vitro* 3D alveolar barrier model would have the potential to become a new enabling platform for studying respiratory diseases and drug efficacy.

## 4 Experimental Section

##### Cell and Tissue Culture

NCI‐H1703 (ATCC, VA, USA) and NCI‐H441 (ATCC), alveolar epithelial cell lines, were cultured in complete medium, RPMI‐1640 (HyClone, UT, USA) supplemented with 10% fetal bovine serum (FBS; HyClone) and a 1% antibiotic/antimycotic solution (HyClone). All cell types were cultured at 37 °C in a humidified 5% CO_2_‐containing atmosphere. MRC5 (ATCC), lung fibroblasts, were cultured in MEM α (HyClone) with 10% FBS and a 1% antibiotic/antimycotic solution. HULEC‐5a (ATCC) were cultured in MCDB 131 medium (Thermo Fisher Scientific, MA, USA) supplemented with 10 mm l‐glutamine (Sigma‐Aldrich, MO, USA), 1 µg mL^–1^ hydrocortisone (Sigma‐Aldrich), 10 ng mL^–1^ human EGF recombinant protein (Thermo Fisher Scientific), 10% FBS, and a 1% antibiotic/antimycotic solution. The cells were subcultured upon growth to a sufficient level of confluence.

The all‐inkjet‐printed alveolar barrier was cultured in the complementary MCDB131 culture medium with 100 KIU (Kallikrein Inhibitor Units) aprotinin (A1153, Sigma‐Aldrich). 500 µL of medium was added to the basolateral side of 12‐mm ‐Transwell with a 10‐µm porous membrane, whereas the apical side was exposed to air during the incubation. The culture medium was replaced once a day. They were cultured at 37 °C in a humidified 5% CO_2_‐containing atmosphere for 14 days. As a comparison to the 3D structured model, the 3D non‐structured model was prepared by homogeneously mixing and printing the same number of cells and the same volume of collagen. The cell‐free collagen model was prepared with the same volume of collagen without cells.

##### Cell‐Laden Ink Preparation for Inkjet Bioprinting

NCI‐H1703‐, NCI‐H441‐, and HULEC‐5a‐laden cell inks were prepared for inkjet printing by suspending cells in complete culture medium in which each cell type had been cultured. The total number of printed NCI‐H1703 cells, NCI‐H441 cells, and HULEC‐5a cells were 8 × 10^4^, 1.6 × 10^5^, and 3 × 10^5^, respectively, which simulated the proportion of cells within the natural human alveoli.^[^ ^5^ ^]^ Cells were detached from a 150‐mm cell culture plate by adding 3 mL of Accutase (Innovative technologies, CA, USA) for 3 min. The cell growth medium was added to neutralize the enzyme activity and the cell suspension was centrifuged. The cell pellet resuspended in medium were filtered through a 40‐µm pore strainer and then counted using an automated cell counter (Countess II FL; Invitrogen, CA, USA) to produce a bio‐ink concentration of 6 × 10^6^ cells mL^−1^.

A collagen ink containing MRC5 cells was prepared by suspending cells in neutralized collagen solution. To prepare neutralized collagen solution, the collagen solution containing 0.375% w/v atelocollagen (Dalimtissen, Korea) in 0.1% acetic acid, reconstitution buffer, and nutrient buffer were mixed at a volume ratio of 8:1:1. Reconstitution buffer consisted of NaOH, HEPES, and NaHCO_3_. The nutrient buffer included powdered DMEM, Ham's F12, and penicillin/streptomycin solution. The final concentration of collagen was 0.3% w/v in solution with a pH of 7.4. To prepare MRC5 cell‐laden ink, cells were detached with Accutase and centrifuged. Cells were counted using the automated counter to produce a bio‐ink concentration of 3 × 10^6^ cells mL^−1^ in 0.3% collagen solution. The total number of printed MRC5 cells on a model were 1.8 × 10^5^.

##### Inkjet Bioprinting System

The bio‐ink was ejected in the form of droplets using the drop‐on‐demand (DoD) inkjet bioprinting system (Jetlab II; MicroFab, TX, USA) based on a piezoelectric printing method including an *x*–*y* motorized stage and a nozzle moving along the *z*‐axis with a high resolution of ±2 µm. An inkjet printhead with an 80‐µm‐diameter nozzle (MJ‐ATP‐01‐80; Microfab) was used. The temperature of ink reservoirs and nozzles in the system was carefully controlled by an integrated cooling system with a refrigerated circulation bath (JSRC‐13C, JS Research, Korea). Parameters related to voltage application were adjusted to control the size and straightness of the drop. A bipolar voltage waveform was applied to the piezoelement via a JetDrive controller (MicroFab) with ±80 V, and rise, dwell, and fall times of ≈10 µs. The velocity of the droplet was adjusted to 3 m s^−1^. The speed and acceleration of the *x*–*y* moving stage were 15 mm s^−1^ and 500 mm s^−2^, respectively. Cell arrays and patterns were printed from monochrome bitmap images. The number of cells to be printed by inkjet printing was adjusted by controlling the number of printed drops, the cell concentrations of bioinks, and the spacing between drops. The cell‐laden inks were re‐suspended by pipetting every 10 min to prevent cells from sedimentation during the printing process.

##### Cell Viability Assay

Cell viability was tested immediately after printing, using the LIVE/DEAD Viability/Cytotoxicity kit (Invitrogen). The cells used in this assay were printed in a 35‐mm cell culture plate filled with 0.5 mL of culture medium to match the original culture conditions. A control group was prepared by manual pipetting. Cells from both control and printed groups were treated with the LIVE/DEAD assay reagent for 30 min. The numbers of stained cells were counted using the automatic cell counter. Cell proliferation was also assessed in both control and printed groups using Cell Counting Kit 8 (CCK 8; Dojindo Laboratories, Japan). A thousand cells used for these assays were printed and pipetted in a 96‐well culture plate filled with 0.2 mL of complete culture medium. Assay reagent was mixed with the cell growth medium at a ratio of 1:10. A total of 0.2 mL of mixture was added to the cells and incubated for 1.5 h. The UV absorbance of each well was measured at 450 nm on days 1, 3, 5, and 7 using a microplate reader (Spark; Tecan, Switzerland).

##### Thickness Measurement by Stylus Surface Profiler

A stylus surface profiler (Dektak XT; Bruker, MA, USA) was used to scan the precise thickness of the alveolar barrier model. The tissue with a porous membrane was cut out from the Transwell. The separated tissue on the membrane was placed on a cover glass and then measured its thickness by continuous stylus tip movement. To minimize the stress on the inkjet‐printed model, the minimal force of the stylus tip was set to 1 mg. Approximately 7 mm of the middle part of the model, which is representative of the overall model thickness, was measured. The thickness of the model can be obtained by subtracting the thickness of the porous Transwell membrane from the whole thickness of this model including the porous membrane.

##### Histological and Immunohistochemical Analysis

For histological evaluation of printed alveolar barrier models, samples were fixed in 4% paraformaldehyde. The samples containing a porous membrane were cut out from the Transwell. The samples were embedded in OCT compound (Leica Biosystems, Germany) and frozen at −80 °C. Serial 10‐µm‐thick sections were obtained using a cryostat (CM1860; Leica Biosystems) and dried at room temperature for 12 h. The tissue sections were stained with Hematoxylin (Mayer's; Dako, CA, USA) and Eosin Y (0.5% alcohol; Merck, Germany) to visualize and compare the structures of the alveolar barrier models under an optical microscope. To observe surfactant secretion of AT II (NCI‐H441), the sections were stained with Alcian Blue (pH 2.5; Sigma‐Aldrich), which enabled specific staining of surfactant, and Nuclear Fast Red (Sigma‐Aldrich), which stained cell nuclei. Images of the stained samples were acquired with a microscope (DM500; Leica Microsystems, Germany).

To evaluate the functional roles of cells and the existence of ECM in each layer, immunohistochemical analysis was performed with tissue slices. Blocking solution was prepared by dissolving 5% FBS and 0.1% Triton X‐100 (Sigma‐Aldrich) or 10% FBS and 0.2% saponin (Sigma‐Aldrich) in PBS. List of antibodies is shown in Table S1, Supporting Information. To confirm the structure of alveolar epithelial cells, Alexa Fluor 594‐conjugated F‐actin probe (1:100, a12381; Thermo Fisher Scientific) was used. Cell nuclei were stained with Hoechst 33342 (1:5000; Sigma‐Aldrich). The images were captured by a fluorescent microscope (Ti‐S; Nikon, Japan) and a confocal microscope (TCS SP5; Leica Microsystems).

##### TEER and Permeability Measurements

Pre‐warmed Dulbecco's phosphate‐buffered saline (DPBS; HyClone) was added to the apical (0.5 mL) and basolateral (1.5 mL) chambers of the Transwell; then, the alveolar barrier model was incubated for 20 min. The TEER value was measured using an EVOM2 (World Precision Instruments, FL, USA) with double chopstick electrodes (STX2, World Precision Instruments). The permeability of the alveolar barrier model was investigated with FITC‐dextran with an average molecular weight of 70 kDa (Sigma‐Aldrich) as a probe for successful permeability. The fabricated alveolar barrier models were rinsed with Hanks’ Balanced Salt Solution (HBSS; Sigma‐Aldrich) twice and transferred to another fresh 12‐well plate, which was filled with 1.5 mL of HBSS. Next, 0.5 mL of HBSS containing 1 mg mL^–1^ of FITC‐dextran was added to the apical side of the Transwell and incubated for an hour. The transferred FITC‐dextran concentration from each lower chamber was determined using a fluorescence multi‐well plate reader (Spark; Tecan) with excitation and emission wavelengths of 490 and 520 nm.

##### Surface Tension Measurement

The drop spreading technique was used to investigate the apical surface properties of the alveolar barrier model. A droplet from a mixture of dimethyl phthalate/1‐octanol 4:1 v/v with 4 mg mL^−1^ methylene blue was deposited on the apical surface of fabricated 3D models with a home‐built microextrusion dispensing system with a 100‐µm‐sized nozzle and a pneumatic type controller (ML‐5000X II, Musashi Engineering, Japan). The values of surface tension were obtained from the ratio between the diameter (*D*_o_) of an initial droplet from the nozzle and the contact diameter (*D*) on the surface.^[^ ^30^, ^31^ ^]^

##### RNA Extraction and qPCR

The fabricated tissues and four types of cells were washed with PBS and centrifuged. Total RNA was extracted from pellets by using RNeasy Mini Kit (Qiagen, Germany) then measured its amount using UV‐Vis spectrophotometer (NanoDrop One). cDNA was synthesized by using oligo dT and High Capacity cDNA Reverse Transcription Kit (Applied Biosystems, CA, USA). Each cDNA reversely transcribed from mRNA was detected using a real‐time PCR system (StepOne Plus, Applied Biosystems) with SYBR Green Master Mix (Applied Biosystems). Sequences of forward and reverse primer pairs are shown in Table S2, Supporting Information. The reference gene, glyceraldehyde 3‐phosphate dehydrogenase (GAPDH), was used to normalize the raw C_T_ (cycle threshold) values.

##### PR8 Influenza Virus Infection and Analysis

For infection of the 3D structured alveolar barrier model by influenza A virus (A/Puerto Rico/8/1934[H1N1], provided by Dr. Adolfo Garcia‐Sastre, Mount Sinai School of Medicine, NY, USA). 10 TCID_50_ mL^–1^ of PR8 influenza virus was infected into the top epithelial surface of the air–liquid cultured alveolar barrier model while being submerged in serum‐free RPMI‐1640 medium for 3 h. Influenza virus‐containing medium was then removed and washed with PBS in both the apical and the basolateral sides of the alveolar barrier model. Alveolar barrier models were then incubated with complete RPMI‐1640 medium for the indicated times. For infection of the alveolar cell lines with PR8 virus, NCI‐H1703, NCI‐H441, HULEC‐5a, and MRC5 cells (1 × 10^6^ cells) were infected with 10 TCID_50_ mL^–1^ of PR8 virus for 3 h with serum‐free RPMI‐1640. Cells were then washed with PBS and incubated with complete RPMI‐1640 medium.

To measure influenza virus replication, type I IFN, and ISGs mRNA expression, total RNA was extracted using RNAiso Plus (Takara Bio, Japan) from the alveolar barrier model or each cell type. The extracted RNAs (1 µg) were reverse‐transcribed with ImProm‐II Reverse Transcriptase (Promega, Madison, WI, USA) using random primers (Promega). Quantitative intracellular viral RNA or cytokine mRNA levels were analyzed using a real‐time PCR system (StepOne Plus, Applied Biosystems) with cDNAs. Sequences of forward and reverse primer pairs are shown in Table S2, Supporting Information. The reference gene, 18S rRNA, was used to normalize the raw C_T_ values.

##### Statistical Analysis

Quantitative data are expressed as means, with error bars representing standard deviation or standard error of the mean. Unpaired two‐sided Student's *t*‐test analysis and one‐way analysis of variance (ANOVA) with Bonferroni post hoc test was carried out to determine the statistical significance of differences between experimental groups using OriginPro 2016 software (OriginLab, MA, USA). Sample size (*n*) and preprocessing normalization of data was given in the corresponding figure legend. In all cases, a *p*‐value of <0.05 was considered to reflect significance (*p<0.05, **p<0.01, ***p<0.001).

## Conflict of Interest

The authors declare no conflict of interest.

## Supporting information

Supporting Information

## Data Availability

Data available on request from the authors.
