# Supplementary material for: All‐Inkjet‐Printed 3D Alveolar Barrier Model with Physiologically Relevant Microarchitecture
Source: Adv Sci (Weinh). 2021 Mar 8;8(10):2004990. doi: 10.1002/advs.202004990 (PMC8132150; doi:10.1002/advs.202004990)
Supplement: Supplementary file 1 — Supporting Information [file ADVS-8-2004990-s001.pdf]

© 2021 Wiley-VCH GmbH

ADVANCED SCIENCE
Open Access

Supporting Information

for *Adv. Sci.*, DOI: 10.1002/advs.202004990

All-Inkjet-Printed 3D Alveolar Barrier Model with Physiologically Relevant Microarchitecture

Dayoon Kang, Ju An Park, Woojo Kim, Seongju Kim, Hwa-Rim Lee, Woo-Jong Kim, Joo-Yeon Yoo, and Sungjune Jung**

Supporting Information

All-Inkjet-Printed 3D Alveolar Barrier Model with Physiologically Relevant Microarchitecture

Dayoon Kang, Ju An Park, Woojo Kim, Seongju Kim, Hwa-Rim Lee, Woo-Jong Kim, Joo-Yeon Yoo, and Sungjune Jung**

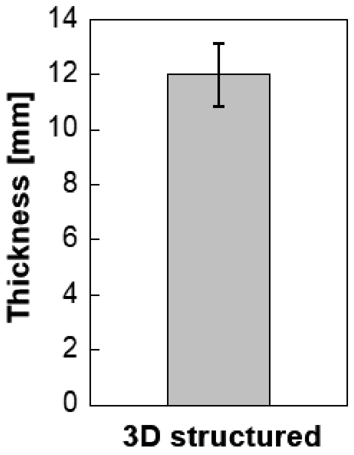

Figure S1. Evaluation of thickness of fabricated 3D structued model The thickness of 3D structured model were accurately measured by a stylus surface profiler before tissue fixation and freezing (n=5).

Table S1. Antibody list

Antibody name	Ratio	Serial number; Company
Anti-laminin	1:1000	ab11575; Abcam
Anti-cluster of differentiation 31 (CD31)	1:1000	ab28364; Abcam
Anti-zonula occludens-1 (ZO-1)	1:1000	61-7300; Thermo Fisher Scientific
Anti-collagen IV	1:1000	ab6586; Abcam
Anti-surfactant protein A	1:500	ab87674; Abcam
Goat anti-Rabbit secondary antibody, Alexa Fluor® 488	1:200	a11034; Thermo Fisher Scientific

Table S2. qPCR primer list

Target gene	Primer sequence (5’ → 3’)
ZO-1 (TJP1)	F: GCGGTCAGAGCCTTCTGATC
	R: CATGCTTTACAGGAGTTGAGACAG
E-cadherin (CDH1)	F: GCTGGACCGAGAGAGTTTCC
	R: GGTGTATACAGCCTCCCACG
Occludin (OCLN)	F: TCAGGGAATATCCACCTATCACTTCAG
	R: CATCAGCAGCAGCCATGTACTCTTCAC
SP-A (SFTPA2)	F: TTGGAGCCTGAAAAGAAGGA
	R: GGCTTGGAGCTCCTCATCTA
SP-B (SFTPB)	F: AGGTGGACTTCCAGCTTTTGAT
	R: CAGCACTTTAAAGGACGGTGTC
α-ENaC (SCNN1A)	F: CTTTGGCATGATGTACTGGCA
	R: GGAAGACGAGCTTGTCCGAGT
β-ENaC (SCNN1B)	F: GAGCCCTGCAACTACCGGA
	R: GCCGAAGGAAGTGCCTTCTC
α_1-Na^+-K^+-ATPase (ATP1A1)	F: AGTTGCAGGAGATGCCTCTGA
	R: TCTCGACGATTTTGGCGTATC
GAPDH	F: CTCCTCTGACTTCAACAGCGACA
	R: GAGGGTCTCTCTCTTCCTCTTGT
Influenza M1	F: TCTGATCCTCTCGTCATTGCAGCAA
	R: AATGACCATCGTCAACATCCACAGC
interferon-β	F: TGCTCTCCTGTTGTGCTTCTCC
	R: CATCTCATAGATGGTCAATGCGG
ISG15	F: CCTCTGAGCATCCTGGT
	R: AGGCCGTACTCCCCCAG
OAS1	F: TCAGAAATACCCAGCCAAA
	R: GAGCCACCCTTTACCACCTT
18S rRNA	F: CGCCGCGCTCTACCTTACCT
	R: TAGGAGAGGAGCGAGCGACC
